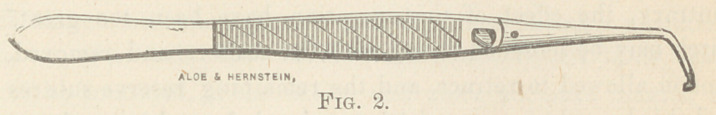# Advancement of the Rectus

**Published:** 1881-09

**Authors:** A. E. Prince


### Original Communications.

---

ARTICLE II.

ADVANCEMENT OF THE RECTUS. By A. E. PRINCE, M.D.*

The operation of advancement of the rectus, from the crude state in which it was first proposed by Guérin (1849), has been improved and rendered more exact by many, among whom may be mentioned the names of Von Graefe, Critchett, Lebreich, Snellen, Knapp, Wecker, Pannus, Agnew, Noyes, Green and others, who have written upon the subject.

Yet the operation has hitherto remained imperfect on account of the difficulty of calculating the result in a given case, and the want of what may be called *adjustability*, rendering the operation incapable of being subsequently modified. It has hence always been regarded as a difficult and unsatisfactory operation rarely employed except in the most aggravated cases.

Though the limit of our space prevents a historic analysis of the progress of the operation, the introduction of Wecker's double-hook, one branch of which is designed to be inserted beneath the tendon, while the other slides down upon it from above, is worthy of special mention, for by thus securing the tendon until after the insertion of the sutures, the execution is freed from danger and greatly facilitated ; though by his peculiar stitch, the tendency to split the muscle—or displace it upward or downward—or cause a slough of the strangulated area between the sutures and the cornea, is so great as to constitute insurmountable objections to the method.

The two pre-requisites of a typical advancement operation, are—1st. Avoidance of the tendency on the part of the stitch to

---

* Reprinted from the *St. Louis Medical and Surgical Journal.*

divide the fasciculii and split the theca of the R. muscle, allowing it to retract, thus compromising or nullifying the effect, or even making the case worse. 2. A desideratum which has formerly received but little or no attention, is that of enabling the operator to *modify at will the effect of the operation after* recovery from the anæsthetic, and when the elastic tension and muscular contractility have returned.

The first of these indications is met by the loop-suture.

The second, by the *double loop suture* as employed in the following operation, which, while it seems to combine the advantages of other operations, avoids their objectionable features.

FIG. 1.

OPERATION.

The patient being asleep and speculum introduced, a fold of conjunctiva over the insertion of the tendon of the muscle to be advanced is grasped with the fixation forceps and elevated. A needle armed with a salicylized silk suture* is passed through

---

* The salicylized suture is prepared by immersing the silk on the spool in a solution of salicylic acid 1 part, alcohol 9 parts, glycerine 1 part; the end of the thread being allowed to protrude through a notch in the cork; thus it can be drawn out and cut off as wanted.

On evaporation of the alcohol, the glycerine retains the acid and keeps the suture damp and pliable. Such suture is no more irritating than the silver, until after the acid is dissolved, and is especially favorable when the cutting out of a suture is objectionable.

the elevated conjunctiva, parallel to and about two or three m.m. from the corneal-margin, after which the needles are cut off, making two loop sutures, as represented in the fig. (a) and (à).

A small opening through the conjunctiva and Tenon's capsule below and opposite the insertion of the tendon to be advanced is then made in the usual manner to admit of the introduction of one branch of Wecker's double hook or appropriate forceps,* which is passed underneath the tendon and drawn tense when the remaining branch is lowered upon the conjunctiva including tendon and cellular tissue.

FIG. 2.

This done, the tendon with conjunctiva is separated from the ball at its insertion, by the introduction of one blade of the scissors through the opening previously made.

Lifting the detached tendon from the ball by means of the forceps, the needles, carrying the *double-loop suture* are introduced from within outward through muscle and conjunctiva, as indicated (b) (b′); the position of the points of puncture depending on the effect desired. This step is facilitated by arranging the needle holder to carry both needles at once, which is important when no anæsthetic is employed, for the requisite time is thereby reduced to about two minutes.

Upon the introduction of the sutures the forceps is to be liberated by separating the contused tendon and conjunctiva, with scissors along the dotted line to the right in the figure, the needles being cut off as indicated.

A subconjunctival division of the opposite tendon having been made at the commencement when necessary, the advancement is accomplished by isolating and twisting sutures (a) and (b) which form secure loops, respectively through conjunctiva and muscle. The parts being cleared of blood, a knot is formed and drawn until the tendon is deemed sufficiently advanced, when it is secured by a simple bow. The conjunctival gap will have simultaneously been closed. After recovery from the anæsthetic,

* For forceps, address Aloe, Hernstein & Co., 300 N. Fourth street, St. Louis, Mo.

and upon the return of the muscular tonicity an examination is made to ascertain the success of the operation. The patient being directed to fix some object, there should be no motion upon alternately covering either eye.

If this be the case the remaining sutures (a′) and (b′), after some hours should be withdrawn. Should the effect not have been sufficient, the sutures (a) and (b) are to be still farther tightened, as much as may be necessary to give parallelism to the eyes, and the knot secured, sometime after which the remaining sutures (a′) and (b′) are to be removed as before. Should, on the contrary, the effect of the operation have been too great, the stitch may be loosened or cut with the scissors and removed, the tendon allowed to retract, and the remaining reserve sutures (a′) and (b′) brought into requisition, twisted, formed into a knot, and sufficiently drawn to make the effect of the operation perfect.

The stitch may be allowed to remain until it becomes loose when it can be removed without pain.

The occasions for performing this operation, though quite numerous, may be naturally arranged in six classes.

1. Paretic and paralytic affections, where the muscular contractility cannot be calculated.

2. In case of over correction from a previous operation either from faulty operation, the muscle having been divided outside of the capsule, or in case of squint due to hypermetropia, the correction of which would have corrected the strabismus. When such eyes are straightened and the person subsequently uses glasses, the eye deviates in the opposite direction as much as it would originally have been corrected by glasses.

3. In case of strabismus of one eye exceeding 15° the muscular relations of the other eye being normal. In such cases one should confine the operation to the deviating eye, dividing one R. and advancing the opposite.

4. When both eyes deviate, amounting together to more than 30° after H. has been corrected. In such cases it is known that simple division of both R. Interni rarely suffices. A preparation for an advancement on the side of the greater deviation should be made, and the strabismus a little over corrected to meet the cicatricial contraction of the two opposing muscles, thus accomplishing in one operation what is seldom a perfect success after several.

5. In case of a slight deviation, where we fear a simple division would produce an over correction. We should employ the advancement stitch to limit the effect of the division.

6. In case of binocular diplopia, this operation on account of its *adjustability*, is rendered especially applicable.

In order to put in practice the above, the reader must be familiar with the method of measuring strabismus by *degrees*, which alone seems the rational method of designating a deviation in the arc of a circle; and besides, the measurement in degrees, as will be conceded, is the easier and more accurate manner of determining the amount of deviation in any case of squint.

The observer should be provided with a perimeter or graduated semicircle, of from 15—18 in. radius; the degrees being numbered from the center toward the extremities and mounted upon a pedestal which will allow of various degrees of elevation, that it may be placed upon the same level with the eyes of different persons. The deviating eye is placed at the center of the diameter and the patient directed to look at the zero of the semicircle at which is placed a small colored disc, for a fixation object. If now a candle be placed immediately behind the zero point, and the observer's eye directly behind this; where no deviation exists, the image of the flame will be seen in the center of each pupil. Should the eye deviate the image will appear at some other point than the pupillary center. While the non-squinting eye continues to be fixed on zero, both candle and eye of the observer are to be simultaneously moved along the arc until the point is found, when the image of the candle flame is seen in the middle of the pupil. This point marks the corneal axis; and since the corneal and optical axes are approximately the same, this is the degree of deviation. When a still more accurate measurement is deemed desirable, it may be accomplished by ascertaining the angle A. It will frequently be found that when the optical axis is directed to zero, the corneal axis may deviate several degrees, which is found by moving the candle until the image is seen in the center of the pupil. The angle of inclination of these two axes, called by common consent among oculists, angle A, is frequently large, causing persons to appear to have an external deviation, when really binocular vision exists. In such cases the optical axis cuts the cornea to the inner side of its zenith, when the angle is called positive, and should such persons have internal strabismus, the angle A of each eye should be added to, and in external, subtracted from the results of the former approximate measurements, which will usually be found sufficiently accurate. For this excellent and simple method we are indebted to Snellen of Utrecht, and Londolt of Paris,* in whose clinics alone the author has seen it employed; and it is hoped that the knowledge of such an admirable device will become more generally disseminated; for accurate measurements are essential to success, whether by the simple division operations or in cases where an advancement is indicated.

A few selected cases from our case-book will illustrate the above theoretical consideration, and serve to show the obstacles met with in some rare and difficult cases.

† No. 1016. Mr. A., aged 45, after correction of H. concomitant S=35°, external motion quite limited. R. E. $V=\frac{20}{30}$, L. E. $\frac{20}{200}$. Seen through ground glass which prevents fixation, both eyes deviate about equally. Divided both internal tendons and introduced the advancement stitch for external R. of L. E.

The external muscles being quite weak found it necessary to advance to a considerable degree.

No. 1143. Master J., aged 12 years, concomitant S. 45° since infancy. No H., L. E. $V=\frac{20}{20}$, R. E., counts fingers at three feet. Division of both internal recti—and advancement of R. E. of the left. No difficulties.

No. 1329. Miss L., aged 18, concomitant alternating S. since 18 months of age, 50°. H=1.5 D $(+\frac{1}{24})$, after the correction of which S=40°.

External motion of L. E. more limited than R. Divided both R. I. and advanced R. E. of the L. E. This not being sufficient, loosened the knot and advanced until perfect. No difficulty.

* Handbuch der Augenheilkunde. Graefe and Saemisch, Vol. III, p 233. The author employs a tangent to a circle in place of the regular perimeter.

† Above abbreviations. H=Hypermetropia; S=Strabismus; R. E.=right eye; L. E. = left eye; V=acuteness of vision; D=dioptric; R. E.=Rectus Externus; R. I.=Rectus Internus.

No. 1414. Miss P., aged 15, internal squint 50° Hn=0, V=$\frac{20}{30}$ for each eye.

External motion much limited in case of each eye, indicating a relative weak condition of both externi. Found it necessary to divide both interni and advance both externi. Cosmetic effect perfect, the latter R. E. being advanced by a second operation. Theoretically both R. E. should have been advanced at the first operation, but the necessity for the second operation is but testimony in favor of the rule—which, had it been followed, would have led to a perfect operation the first time.

No. 1370. Madame D., aged 42. When a child had both R. I. divided for internal S. In place of the tendon, the muscle was divided outside of the capsule of Tenon, and both retracted in the orbit. The external recti operating without any counterforce, an extreme external deviation was the result, amounting to 60° (35° in one eye and 25° in case of the other). It was deemed advisable to attempt an operation, though without much expectation of success. The external recti were accordingly divided, and to increase the liberty toward internal motion, the capsule was split meridianally above and below the muscles which were found to be exceedingly strong. No attachments were found internally, and no muscular fibers. Nothing except the most atrophied conjunctiva. Into this the double-loop suture was placed and the knot tightened to its limit. The temporary result was admirable. For a week there remained a slight internal strabismus which finally became converted into a slight deviation outward (15°), for which a second operation was not considered advisable. This case taught the important lesson exemplified in the next case, viz., that when it is a case of total paralysis, or due to complete separation of the muscle from the eye, the opposite muscle should be divided entirely outside of the capsule of Tenon, that it may be free to retract in the orbit, and exert no more influence on the eye than its paralyzed opponent. When such eye is straightened, we can of course expect only a limited amount of motion.

No. 1546. Mrs. B., *colored*, aged 26, congenital paralysis of third pair, implying loss of power over the superior, inferior and internal rectus, the inferior oblique and levator palpebræ superioris, which last was but partially affected.

The result of this is an external deviation of each eye of 50°, making a combined external strabismus of 100° with immobility of the eyes.

But a small portion of the pupil could be seen in the extreme external canthus of each eye, and when vision was attempted the head was held to the right or left, according to which eye was employed in vision, this being $\frac{20}{60}$ with each eye.

This being the most extreme case which had come to our notice, it gave us an excellent opportunity to test the merits of our operation, which worked according to programme, as will be shown by a photograph illustrating the condition, before and after the operation, and which will be mailed to any one upon receipt of stamp. The condition of partial ptosis remains to be corrected, the photographic views having been taken during artificial suspension of the lids.

From the above it will appear that the range of usefulness of an adequate advancement operation is by no means small, embracing some low and all the high degrees of strabismus, and especially such desperate cases as the above, and when we consider the small proportion of extreme cases heretofore attended by complete cosmetic success, and the large number requiring repeated operation, no apology will be necessary for bringing to the notice of the profession an operation whose *facility of execution*, *security* and *adjustability*, it is conceived, will assist in placing the correction of strabismus upon a basis approximating universal success.

## Figures and Tables

**Fig. 1. f1:**
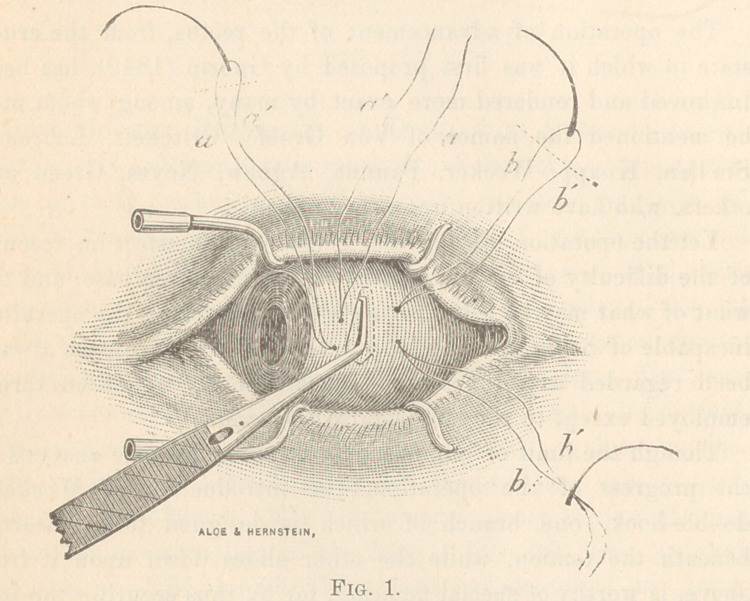


**Fig. 2. f2:**